# Study of mandibular growth in patients treated with Fränkel´s functional regulator (1b)

**DOI:** 10.4317/medoral.17958

**Published:** 2012-05-01

**Authors:** Juan J. Alió-Sanz, Eyleen Kato, Jose Lorenzo-Pernía, Carmen Iglesias-Conde, Alejandro Iglesias-Linares, Enrique Solano-Reina

**Affiliations:** 1 DDS, MS, PhD. Professor of orthodontics, Complutense University of Madrid, Spain; 2 DDS, PhD. Complutense University of Madrid, Spain; 3 DDS, PhD.Private practice, Madrid, Spain; 4 DDS, PhD. Private practice, Orense, Spain; 5 DDS , MSc, PhD. Lecturer Masters Programme in Orthodontics and Dentofacial Orthopaedics. School of Dentistry. University of Seville, Spain; 6 DDS, MSc, PhD. Chairman of orthodontics, University of Seville. Spain

## Abstract

Objectives: The purpose of this study was to assess mandibular growth in patients with Class II division 1 malocclusions when treated with Fränkel´s functional regulator 1b. Study Design: The treatment group was made up of 43 patients that were divided into two groups: prepubescent (n:28), and pubescent (n:15). The control group included 40 patients who did not receive any kind of treatment and were likewise divided into a prepubescent group (n:19), and a pubescent group (n:21). A computerized cephalometric study was carried out and superimpositions were done in order to assess the antero-posterior, vertical and rotational movements of the mandible. A two-way ANOVA with interaction was done to compare the changes between the control group and the treatment group, while the Student t for independent samples was used to compare each age group. Results: The Gnathion and Gonion points showed significant differences in the whole sample (p<0.001) as well as in the prepubescent (p<0.001) and pubescent groups (p<0.05). Rotational changes of the mandible measured using the facial axis and mandibular plane showed no statistical differences between both groups (p>0.05). Conclusion: The results show that the FR produces vertical orthopedic growth in the mandible but not horizontal growth compared to non-treated Class II-type I malocclusion patients. No rotational changes were found in the mandible, but we did record mandibular growth along the inclination of the facial axis.

** Key words:**Fränkel appliance, orthodontics, functional appliances, mandibular growth.

## Introduction

Fränkel´s functional regulator (FR) started out as a therapeutic option for the correction of Class II malocclusions in which there is a deficit in mandibular size (1,2). One of the main characteristics of this appliance lies in that it takes the oral vestibule as the base of treatment intercepting the aberrations of the muscular function. Therefore, it acts as a vestibular-lingual appliance whereas other functional appliances act within the dental arches (3).

Most studies done with FR have admitted to an effect, whether it be structural or positional, on mandibular growth (4,5). Rodrigues et al. (6,7) report a statistically significant increase in mandibular length. Other authors (4) have obtained different results and have ascribed the main effect on the mandible to a change in its position or simply to dentoalveolar effects. When we talk about positional changes of the mandible we are referring to either a clockwise or counterclockwise rotation. Some authors have obtained results that support the claim the FR produces a clockwise rotation of the mandible (8). This effect could be negative in the treatment of Class II malocclusions, especially in dolichofacial patients. However, a counterclockwise rotation of the mandible is often considered an extremely beneficial effect for the treatment of these malocclusions (9). There are also studies that deny any kind of positional change in the mandible with FR (10). As far as mandibular size is concerned, some authors indicate a significant increase in those patients treated with a FR (11,12). Nonetheless, while most studies do find greater growth, the differences are not significant (10,12-17).

The aim of this study is to assess the changes in size, both sagittally and vertically, and possible rotational effects that treatment with FR type II may have on the mandible using cephalometric parameters and superimpositions that compared with non-treated Class II-type I malocclusion patients.

## Material and Methods

Two groups of patients who had mandibular bone Class II were chosen to participate in this study. One group was treated with the Function Regulator type II and the other group acted as the control group.

We used the following selection criteria for the treatment group (Group I): Class II division 1 malocclusions with mandibular retrusion and a convex profile; an ANB or convexity equal to or greater than 5; a brachyfacial, mesofacial or mesodolichofacial pattern; and they must have undergone orthopedic treatment exclusively with a functional regulator type II. Furthermore, none of the subjects showed signs of having any craniofacial or dental abnormalities and all of them had undergone treatment between the ages of 8 and 14. Following these criteria, the treatment group was made up of 43 patients (18 boys and 25 girls). The average age, in this group, when starting treatment was 9 years/9 months and finishing at 11 years/6 months. This sample was divided into a prepubescent group (8-11 years; n:28), and a pubescent group (12-14 years; n:15) in order to compare the different growth stages. The functional regulator was made according Fränkel`s design (1,3). The constructive bite was done in neutrocclusion with a height of 2-4 mm. The patients were treated for 1 year/6 months on average. Instructions were given to use the appliance for 1 hour a day for the first 15 days, 3 hours a day for the next 15 days, then in addition to the three hours during the day to wear the appliance at night for one month and finally, 60 days after starting, to use the appliance all day and night.

The control group (Group II) included 40 patients (22 boys and 18 girls) with the same malocclusion but who had not received any kind of treatment. These patients refused treatment but were admitted to take part in the craniofacial growth study done by the Department of Orthodontics of the Complutense University of Madrid. The average age at the time of the initial x-ray was 10 years/2 months and at the time of the final x-ray, 13 years/1 month. This group was also divided into a prepubescent group (n: 19) and a pubescent group (n: 21). We used the same selection criteria for this group as we did for Group I. All of the patients in both groups had been born in Spain or were born of Spanish parents.

**Figure 1:**
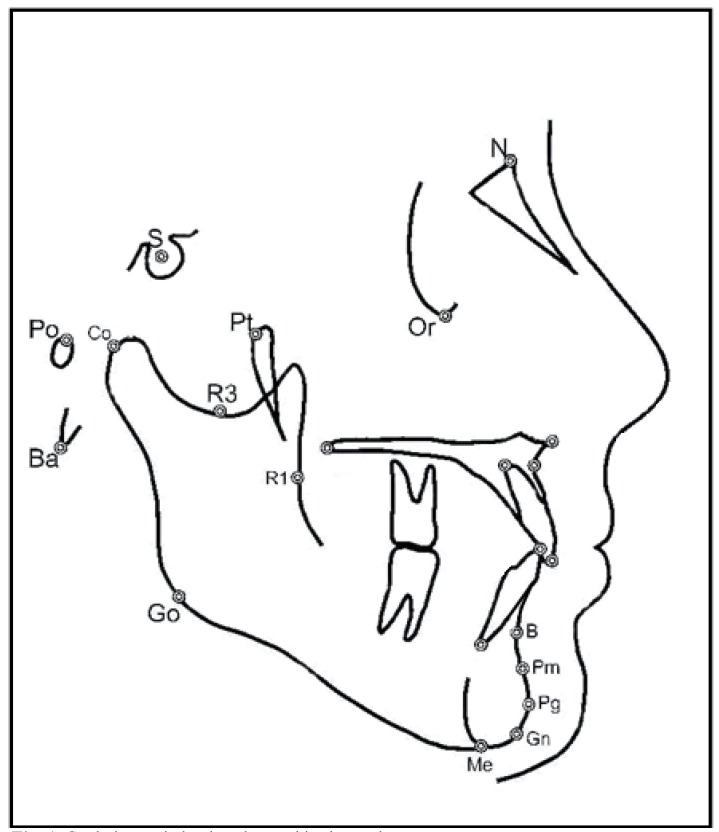
Cephalometric landmarks used in the study.

A lateral x-ray of the cranium was taken of all the patients at the beginning and at the end of treatment. All of the x-rays were taken with the same machine: a Siemens Palomex OY, with a magnification index of 1:1.25. The x-rays were digitalized using an Epson Expression 1680 scanner and cephalometric tracings were done with Nemotec Dental Studio v.2.0.0.1 orthodontic software with reference to the following landmarks: N (nasion), S (sella turcica), Ba (basion), CC (pterygomaxillary), Pt (pterygoid), Po (porion), Or (orbital), A (point A), ANS (anterior nasal spine), PNS (posterior nasal spine), Co (condylion), B (point B), Pg (pogonion point), Go (gonion point), Gn (gnathion point), Me (menton point), Pm (suprapogonion), Point R1, Point R3 (Fig. 1).

**Figure 2:**
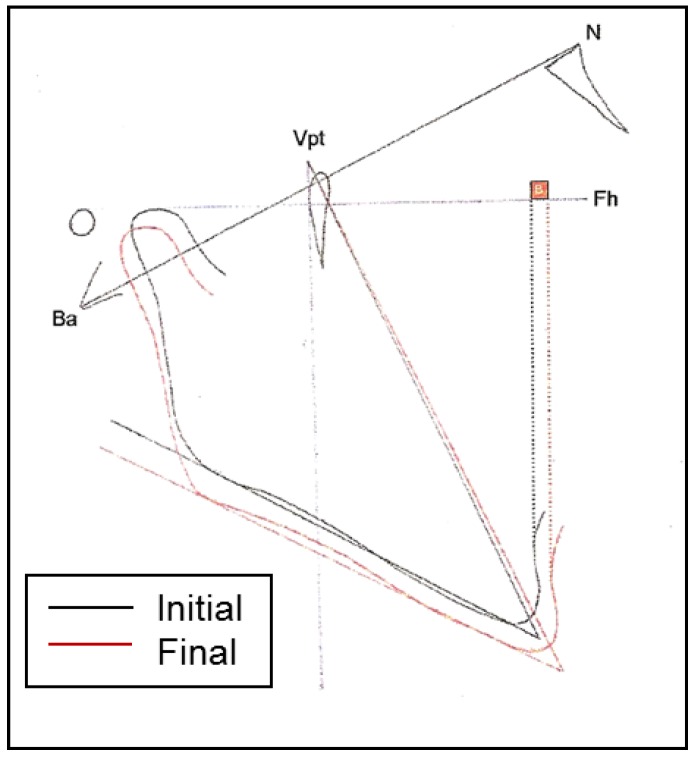
Type of cephalometric superimposition of the initial and final x-rays in both groups in the Ba-N plane, using the Cc as the fixed point.

With these landmarks we performed linear and angular measurements as follows: SNB angle: angle formed by the Sella-Nasion (S-N) and Nasion-Point B (N-B) planes; SND angle: angle formed by the Sella-Nasion (S-N) y Nasion-Point D (N-D) planes; Distance from pogonion to nasion perpendicular to frankfurt (Pg-N): distance between the Pogonion (Pg) and a perpendicular to Frankfurt traced from the nasion (Na); Facial depth (N-Pg/FH): angle formed between the facial plane and the Frankfurt plane. Where the facial plane (N-Pg) is formed by joining the nasion and pogonion points; Mandibular body length (XI-Pm): Distance between the XI point and the Pm point; Effective mandibular length (co-gn): Distance between the highest and furthest back point of the condyle (Co) and the gnathion point (Gn); Antero-inferior facial height (Ena-Me): distance between the anterior nasal spine (ANS)and the menton (Me); Facial axis (Pt-Gn/B-N): angle formed by the facial axis (Pt-Gn) and the Basion – Nasion plane. Posterior facial height (Go–Cf): distance between the Cf point and the Go point; Gonion angle (Go): bisection of the posterior plane of the mandibular ramus and the plane of the lower edge othe the mandible; Mandibular plane angle (Go-Gn/SN): angle formed by the Ricketts mandibular plane and the Frankfurt plane. In addition, a superimposition was done of the initial and final x-rays in both groups in the Ba-N plane, using the Cc as the fixed point (Fig. 2). Two reference planes were traced, the Frankfurt plane and the pterygoid vertical plane. If the planes did not coincide in the superimposition we used the bisector of the two. In the Frankfurt plane the projections of horizontal movement of point B and the Pogonion were measured, with a positive value assigned when the final projection was more forward than the initial one. In the pterygoid vertical plane, projections of the vertical changes in the Condylion, Gonion and Gnathion were measured, with a positive value given when the final projection was lower than the initial one. Mandibular rotation was assessed measuring the initial value and final value of the facial axis as well as the mandibular plane, with a positive value given when the final position showed a clockwise rotation.

All of the cephalometries were traced by two researchers using the same criteria. In order to avoid tracing errors, the general directives for this type of studies were followed (18).

Statistical analysis

The statistical analysis was done with the program S.A.S., version 8.2. First, descriptive statistics were done in order to determine the arithmetic mean, standard deviation, percentiles and rank of each variable for both of the groups studied, for each sex and for each age group (prepubescent and pubescent). Then analytical statistics were obtained using the following tests: two-way analysis of variance (ANOVA) with interaction and Student’t for independent samples with a 0.05 confidence level. The analysis of variance was used to determine the behavior of each variable over time in each group. The Student t was used to compare each variable between group I and group II and between each age group.

## Results

Mandibular measurements.

In the overall analysis of the whole sample, the only parameters that showed any significant differences were mandibular length (Co-Gn) and lower anterior face height ( Table 1). Both measurements were greater in the group treated with the functional regulator. When we compared the prepubescent group with the pubescent group we found that the differences in these two lengths only showed up in the latter. Furthermore, the facial depth angle, which did not reveal any statistically significant differences when we analyzed the whole sample, did show statistical differences when we compared the two age groups. The angle was less pronounced in the prepubescent group treated with the functional regulator ( Table 1).

**Table 1:**
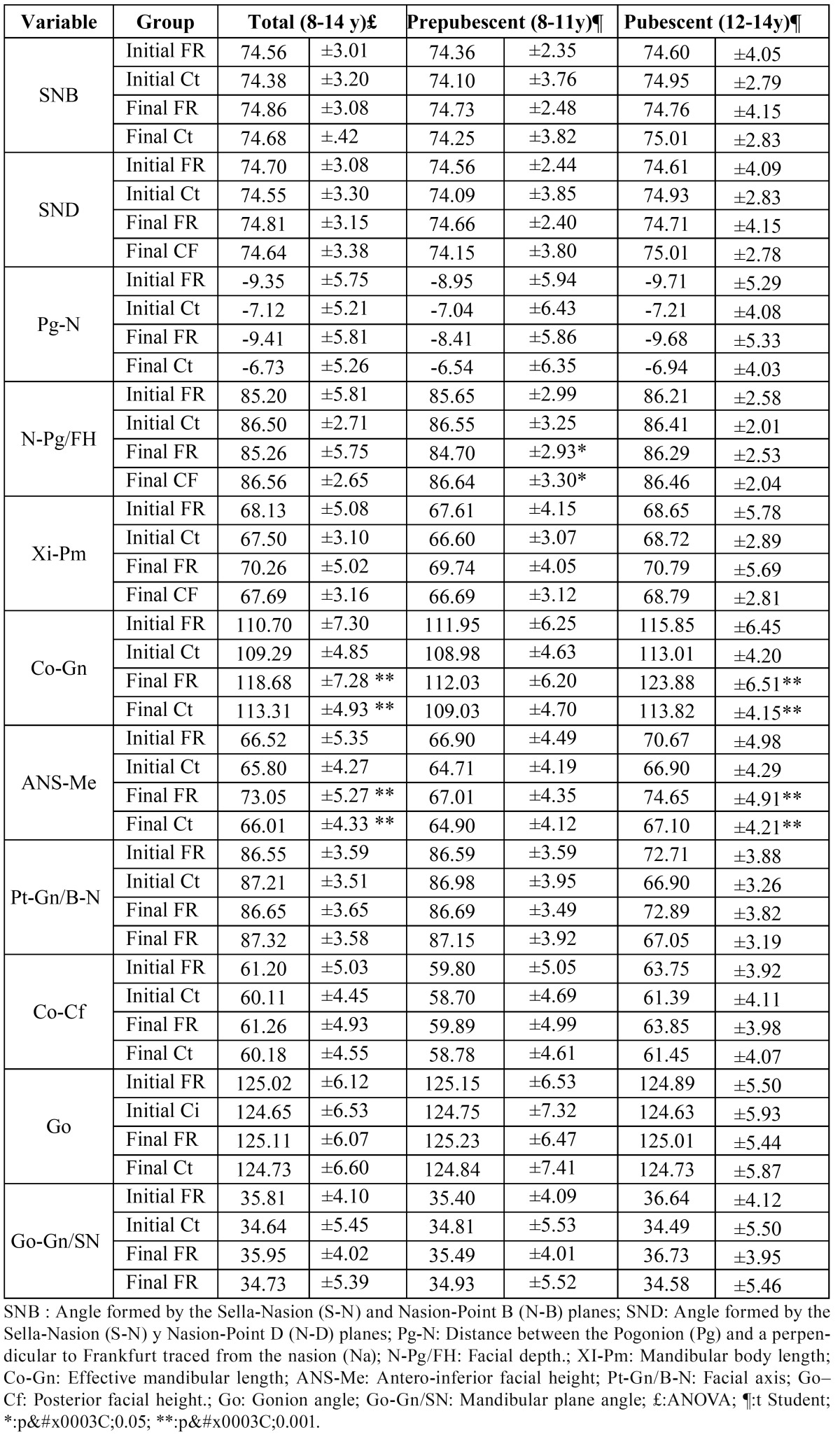
Mandibular measurements.

Comparison of mandibular measurements according to sex.

No statistically significant differences were found in the variables according to sex between the FR group and the control group in any of the eleven cephalometric parameters studied ( Table 2).

**Table 2:**
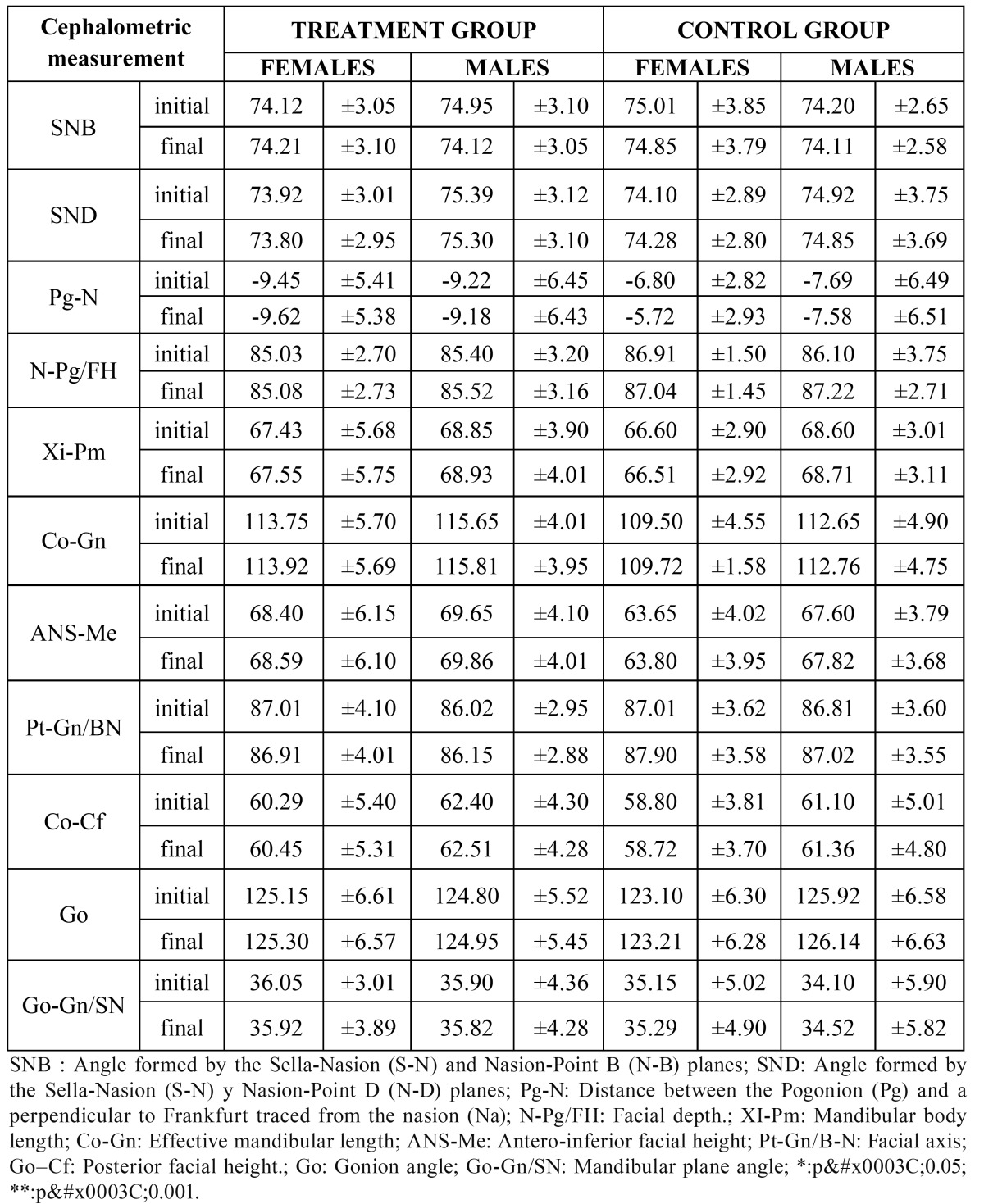
Comparison of cephalometric measurements of the mandible according to sex.

Cephalometric superimpositions.

The sagittal changes in the mandible were studied by analyzing the superimpositions of point B and the Pogonion. The treatment group always showed more movement than the control group at these two points. However, the differences between each group were never significant. It is also important to point out that greater growth of both points occurred in the pubescent group ( Table 3).

**Table 3:**
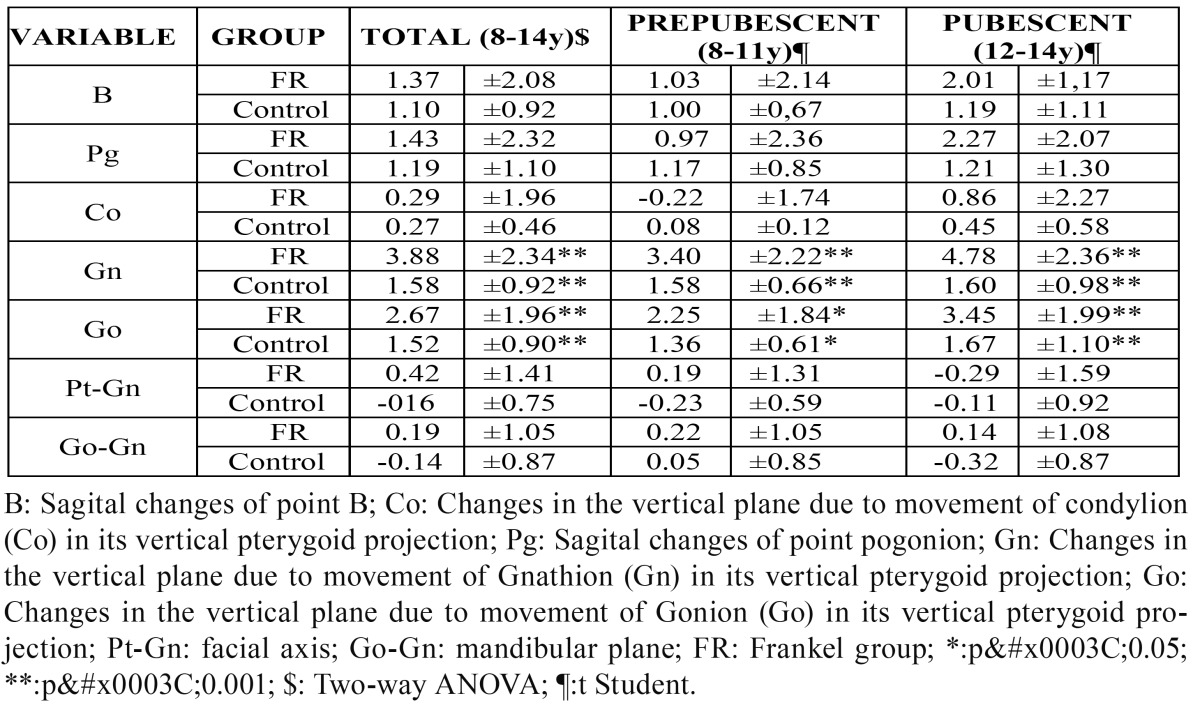
Superimpositions of the mandible.

The changes in the vertical plane were assessed by measuring the movement of three basic points: the condylion (Co), Gnathion (Gn) and Gonion (Go) in their vertical pterygoid projection. The Gn and Go points showed significant differences in the whole sample as well as in the prepubescent and pubescent groups ( Table 3).

The average descent of the Gn point in the treatment group was 3.88 mm, while that of the control group was 1.88 mm. The average descent of the Gonion point was 1.67 mm and 1.52 mm, respectively. Therefore, both points underwent a greater descent in the group treated with the FR. However, no statistically significant differences appeared in the rotational changes of the mandible measured using the facial axis and mandibular plane ( Table 3).

Comparison of superimposition variables according to sex.

We found no statistically significant differences between the males and females of the treatment and control groups in any of the measurements taken of the cephalometric superimpositions ( Table 4).

**Table 4:**
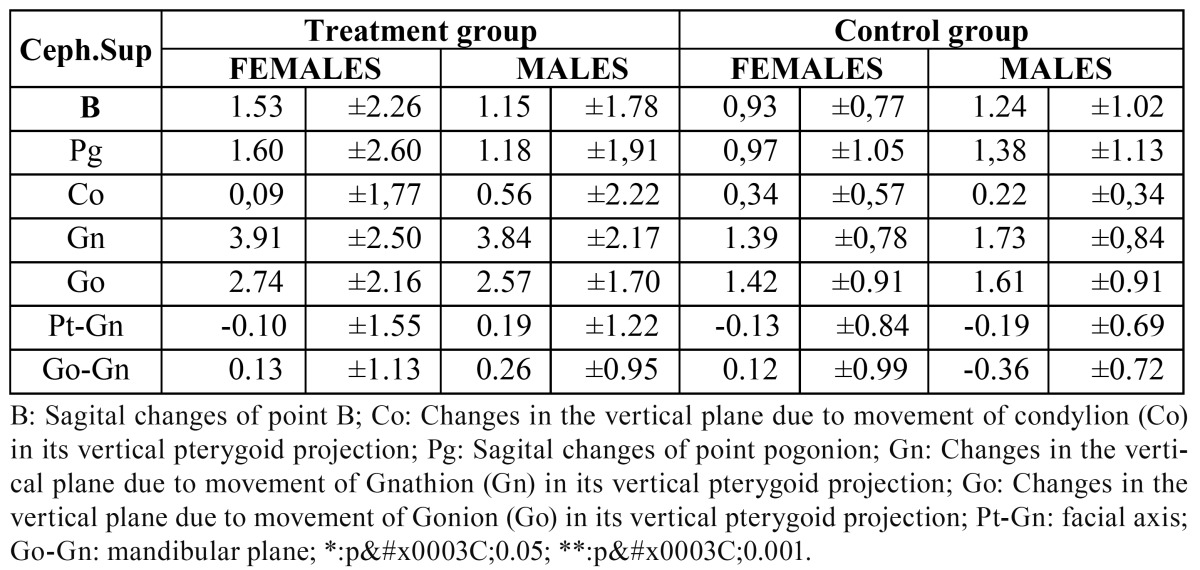
Comparison of the superimpositions of the mandible according to sex.

## Discussion

Sagittal changes in the mandible

None of the measurements which relate the mandible to the cranial base indicated the presence of significant differences between both groups in the study.

The SNB angle, more than the SND angle, is widely used to place the mandible in an antero-posterior position with respect to the cranial base. In this study, as is mentioned above, no significant differences were found between the treatment group and the control group. This would lead us to believe that the functional regulator has no effect on mandibular growth. However, it is important to assess the other measurements in the other planes to reach a final conclusion.

The absence of changes in the SNB in the FR group with respect to the control group is explained by many authors. Rodrigues et al. (6,7) defend the idea that the SNB changes are due to the vestibulization of the lower incisors. This inclination can be a factor which contributes to the erroneous interpretation that there is no mandibular growth. This idea is supported by Adenwalla and Kronman (19), who go on to explain that the positions of point B and point D can be influenced by orthodontic and/or orthopedic treatment. The SNB/SND angle can change due to mandibular rotation in either a hypo- or hyperdivergent direction. A hyperdivergent rotation can reduce the SNB/SND angle while a hypodivergent rotation can increase it. The SND/SNB angle can also increase because of mandibular growth.

Some authors (6,7,20) relate the small change found in the SNB to an increase in lower face height. McNamara (21) has shown that every millimeter of growth in the lower vertical dimension hides a millimeter of growth in mandibular length due to a down and back rotation of the menton. McNamara et al. (15,21) also find that mandibular length is not always manifested in a forward movement of a point in the menton. The changes in the horizontal position of the menton vary inversely to how much the antero-lower face height has increased. McNamara (21) has shown the relation that exists between the increase in the vertical dimension and the antero-posterior position of the menton. The maximum anterior repositioning of the menton is reached by an increase in mandibular length without an increase in antero-lower face height. Therefore, a significant increase in mandibular length can be hidden by an increase in lower face height.

Our results concerning the SNB, Pg-N and facial depth measurements are supported by many publications (20,22-24). Other studies, however, describe quite different results showing a significant forward movement of the mandible (15,24-27). The results reached with respect to mandibular size (Co-Gn) support the idea that mandibular growth increased more in the group treated with FR–especially in those patients between the ages of 12 and 14 (pubescent group).

The superimpositions done of the Pogonion and point B do not indicate any significant sagittal change in the menton when using the FR appliance, although they do show a greater average forward movement in the FR group of 0.24 mm/year and 0.27 mm/year respectively. Likewise, in the superimpositions done on the SN/Nasion axis, some authors (10) find anterior horizontal growth of 0.20 mm in patients after having used the functional regulator for one year. In his superimpositions, Nelson et al. (22) did not find any horizontal movement of the Pogonion in the treatment group when compared to the control group. In contrast, other authors (12,13) find a forward movement of the Pogonion point of 1.50 mm and 2.60 mm respectively. Similarly, Remmer et al. (11) found point B to have moved forward 1 mm, which was not significant with respect to point S.

Vertical changes in the mandible

The results of the superimpositions of the Condylion point indicate that the condyle stays constant since it does not show any differences with respect to the control group. However, here we must point out the elevated standard deviation in the treatment group which indicates the great individual variability of the sample after treatment with the functional regulator.

Other studies in which superimpositions were carried out but which used other frames of reference also find no significant vertical movement of the condyle with respect to the control group. Along these lines are the studies done by Nelson et al. (22) and Creekmore and Radney (23). The latter finds a total growth of 1.1 mm more than in the control group. Hamilton et al. (25) also did a study in which a tomographic analysis was done on a sample treated with the functional regulator. They find that the functional regulator causes a very slight downward and forward movement of the condyle in the glenoid fossa. However, these changes are very small, between 0.2 and 0.3 mm. In any case, the changes were not statistically significant. The only study that refers to a significant descent of the condyle was done by Falck and Fränkel (13) in their superimpositions in the transverse axis of the occipital frame of reference. These authors find the group treated with the functional regulator, and only having a big forward movement of the mandible, experienced a descent of the condylion of 0.14 mm, which was significantly greater than that of the control group.

The superimpositions done to assess the vertical movement of the gnathion show a significant descent - up to 2.3 mm/year in the FR group with respect to the control group. Some authors (23) also found a descent in the menton of as much as 8.5 mm when using the functional regulator. This was significantly greater than that of the control group which was 6.1 mm. In their superimpositions in the transverse axis of the occipital frame of reference, Falck and Fränkel (13) find that the Gnathion in the group treated with the functional regulator descended significantly more than in the control group by 1.13 mm.

The superimpositions done to assess the vertical movement of the Gonion point show a significant descent of more than 1.15 mm/year in the treatment group than in the control group. However, Nelson et al. (22) did not find significant movement of this point.

The interpretation of the measurements as a whole would indicate a tendency for the mandible to descend with the use of the functional regulator.

Rotational changes in the mandible

Neither the superimpositions of the facial axis nor those of the mandibular plane showed significant changes. In other words, there is a tendency to maintain the direction of growth. Likewise, Nielsen (10) does not record changes in the mandibular angulation when using the functional regulator. However, other authors (28) found a significant reduction in the facial axis in his superimpositions of the Fh-VPt. Falck and Fränkel (13), in their superimpositions of the mandibular plane, find a significant increase in that plane showing a tendency to a clockwise rotation, while the control group had a counterclockwise rotation seen in a reduction of the mandibular angle.

Measurements related to mandibular size

The length of the mandibular body (Xi-Pm), while not showing significant differences between the two groups included in the study in any of the age groups, is seen to grow more than 2 mm longer in the FR group when considered over time. It is important to point out that both groups started with a similar mandibular length and that this showed a considerable, although not significant, increase in the treatment group.

The study carried out by Schulhof and Engel (28) supports our findings. This author finds a growth of 3.4 mm of the mandibular body but statistically there were not any significant differences compared to normal growth. Other authors come up with similar results using the Gonion-Pogonion measurement (16,22,27). In contrast to our results, other researchers have found greater growth in the mandibular body (6,7).

The effective mandibular length (Co-Gn) indicates global growth throughout the treatment period of 7.98 mm in the FR group as opposed to 4.02 mm in the control group. The differences were statistically significant. It is important to emphasize the fact that the initial mandibular lengths were similar in both the FR and control groups.

These results lead us to defend the existence of additional mandibular growth as a result of using the functional regulator. Other studies support this idea, such as those done by some authors (6,7,16,20) which note growth of 3.9 mm, 4.3 mm and 4.6 mm, respectively, in those groups treated with the functional regulator. At the same time, there are other studies that do not find any indication of mandibular growth (19,24,29).

When we assessed the effective length according to age groups in our study, we found the greatest changes in the pubescent group. That means the time at which treatment is done could influence the amount of mandibular growth. Some authors (19) believe that the pubescent period, approximately at the time of changing from mixed to permanent dentition, is when we can best take advantage of growth in order to correct Class II. At this time, it would seem, there is a downward and forward growth increase. According to these authors, the best results are obtained when growth is most active.

McNamara et al. (16) mentions that the increase in mandibular length of 4.3 mm/year found in the group treated with the functional regulator cannot be completely accounted for by the forward movement of the mandible, which was 1.2 mm. They associated these differences in position with an increase in the vertical dimension produced by the appliance. Falck and Fränkel (13) similarly affirm that mandibular length was greater with the functional regulator. However, they observed that the Pogonion did not undergo any sagittal changes. This led these authors to believe that the changes in the pogonion were downwards. Consequently, the lower face height increases, as can be seen in the increase of both the mandibular plane and the Gonion angle.

Measurements related to the direction of growth

A significant increase in lower face height was found in our study in those patients treated with the functional regulator. Many other studies confirm these results (24,26,27). Courtney et al. (30) attribute this increase of the vertical dimension to the eruption of mandibular molars. Maxillary molars do not play an important role because the appliance has extensions to prevent the over-eruption of maxillary teeth. Some authors (19) considers that the increase in lower face height is due to alveolar growth and/or to the eruption of subsequent teeth. These authors believe the increase in the vertical dimension can also be due to a subsequent rotation of the mandible. These observations, however, contradict the results published by Righellis (17) and Rodrigues et al. (6) who did not find an increase in this dimension.

McNamara et al. (15) state that an increase in lower face height can be masked by an increase in mandibular length. An antero-posterior change in the position of the menton varies inversely with the increase in the lower face height produced by the treatment.

Some research (19,20) has found that, despite the increase in anterior face height, there is not a corresponding increase in the mandibular plane. These results are similar to those in our study. Some authors (15,20) understand this as the result of similar increases in anterior and posterior face height. This finding is possibly related to the opening of the posterior bite which occurred when the mandible moved forward in the group treated with a functional regulator and as a result there was an eruption of molars. Others (19) add that this behavior is due to an increase in mandibular ramus height.

The facial axis, as in other studies (20,26), does not show any significant differences between the groups studied. Nonetheless, some authors (28) find that the functional regulator tends to produce a clockwise rotation of the mandible. No differences were found in our assessment of the mandibular plane (Go-Gn/SN) between each of the groups studied. No differences between the treatment group and the control group were found in other studies either (22,25). However, Creekmore and Radney (23) found a significant increase in the mandibular plane in the treatment group of 1º, while that of the control group was recorded as a decrease of -1º.

Adenwalla and Kronman (19) write that the increase in mandibular ramus height in the group treated with the functional regulator could keep the mandibular plane stable in spite of the increase in lower face height. Similarly, Rodrigues et al.(6) consider that the absence of changes in the mandibular plane after using the functional regulator could have to do with the interrelationship between the anterior vertical dimension and the posterior vertical dimension.

So, bearing in mind all of the measurements we can deduce that the functional regulator pushes the mandibular body parallel to itself following the facial axis. On average the mandibular plane and the facial axis are not affected by the treatment.

## Conclusions

>1. An increase in mandibular size is observed during treatment with the functional regulator. This increase has no effect in the sagittal plane, but it does in the vertical plane.

2. No rotational changes in the mandible were seen in patients using the functional regulator.
